# Fatal Herpetic Hepatitis in Pregnancy

**DOI:** 10.1155/S1064744994000177

**Published:** 1994

**Authors:** Stephen Chatelain, D. Edward Neumann, Samuel M. Alexander

**Affiliations:** 1 Maternal Fetal Medicine Section Woman's Hospital Baton Rouge Louisiana State University School of Medicine New Orleans LA USA; 2 Department of Obstetrics and Gynecology Louisiana State University School of Medicine New Orleans LA USA; 3 Mercy-Baptist Hospital 4429 Clara Street Suite 540 New Orleans LA 70115 USA

## Abstract

*Background:* Disseminated herpetic infections during pregnancy have been reported in the literature.

*Case:* This case presentation describes a pregnant patient who presented with fever, elevated liver enzymes, and upper abdominal tenderness and succumbed from fulminant herpetic hepatitis.

*Conclusion:* Early diagnosis and treatment are essential because of the high mortality rate.


Infectious Diseases in Obstetrics and Gynecology 1:246–248 (1994)
© 1994 Wiley-Liss, Inc.

### Fatal Herpetic Hepatitis in Pregnancy

**Stephen Chatelain, D. Edward Neumann, and Samuel M. Alexander**

*Maternal Fetal Medicine Section, Woman's Hospital, Baton Rouge (S.C., D.E.N.), and Department of Obstetrics and Gynecology, Louisiana State University School of Medicine, New Orleans (S.M.A.), LA*

Abstract

***Background:*** **Disseminated herpetic infections during pregnancy have been reported in the literature.**

***Case:*** **This case presentation describes a pregnant patient who presented with fever, elevated liver enzymes, and upper abdominal tenderness and succumbed from fulminant herpetic hepatitis.**

***Conclusion:*** **Early diagnosis and treatment are essential because of the high mortality rate.**
© 1994 Wiley-Liss, Inc.

Key words

Herpes simplex virus, thrombocytopenia, pregnancy

### CASE REPORT

The patient was a 20-year-old white female, $G_1$ $P_0$, with an intrauterine pregnancy at 32 weeks gestation by ultrasound examination performed in the referring hospital on October 14, 1991. The patient was seen by her primary physician in the emergency room at the referring hospital 1 week prior to transfer. She was diagnosed with a urinary tract infection and was treated with Septra. The patient continued to experience a febrile course and was admitted to the referring hospital where her temperature was found to be 39.6°C. Her blood pressure was 90/60 and her pulse was 98.

The patient's medical history was negative for diabetes mellitus, hypertension, asthma, hepatitis, and thyroid and kidney disease. She denied the use of tobacco, alcohol, and illicit drugs.

The initial physical examination revealed a patient who was not icteric and not dehydrated. Right upper quadrant tenderness was found. Mild uterine tenderness was also noted. Occasional uterine contractions were noted. Her cervix was 1 cm dilated and 25% effaced with intact membranes.

Initial laboratory values showed a white blood cell (WBC) count of 4,000 with 64% polyps, 18% bands, and 12% lymphocytes. The platelet count was 124,000, and hematocrit 36.8%. Liver enzymes showed an ALT of 1,062, ALP of 204, AST of 2,031, and lactate dehydrogenase (LDH) of 1,918. A liver ultrasound was obtained and did not show any gallstones. Hepatitis B surface antigen was negative. The patient's prenatal laboratory results showed her to be human immunodeficiency virus (HIV) negative. Differential diagnosis included hemolytic uremic syndrome, acute fatty liver, and hepatitis.

The patient was treated with magnesium sulfate tocolysis for the uterine contractions and was transferred to the tertiary care hospital on December 22, 1991. On her arrival at the tertiary care hospital, her temperature was 38.3°C, her pulse was 108, and her blood pressure was 110/60. A repeat physical examination showed the lungs to be clear. Abdominal palpation showed definite right upper quadrant tenderness. The lower abdomen and uterus were non-tender to palpation. The patient

Address correspondence/reprint requests to Dr. Samuel M. Alexander, Mercy-Baptist Hospital, 4429 Clara Street, Suite 540, New Orleans, LA 70115.

Received November 23, 1993

Accepted March 4, 1994

was alert and oriented. Her cervix was 1 cm dilated, long, and posterior.

Laboratory examination showed the WBC count to be 2,800, platelets 86,000, PT and PTT within normal limits, fibrinogen 386, and bleeding time 4.5 min. A urinalysis showed 2+ ketones but no evidence of bile, blood, nitrites, or leukocyte esterases. Two to 4 WBCs were noted with a rare red blood cell (RBC). Repeat chemistry panel showed an AST of 4,053 and an LDH of 12,255. Bilirubin was 1.0. Bacterial cultures were obtained of the urine, blood, cervix, and amniotic fluid.

An internal medicine consultation was requested soon after the patient's arrival at the tertiary care hospital.

The assessment of the patient was 1) fever of unknown origin; 2) epigastric pain, rule out gastritis, gastric reflux, and hepatic enlargement; 3) thrombocytopenia; and 4) elevated liver enzymes. The patient was started on ceftriaxone and tobramycin. Magnesium sulfate was continued at 2 g/h. TORCH titers were ordered. Twelve hours after her admission, the patient's physical examination had not changed. The patient complained of hunger and thirst. She was no longer contracting. The cervix was unchanged. The uterus was non-tender.

Ultrasound examination showed the fetus to be consistent with 32 weeks gestation. A liver and spleen ultrasound did not show enlargement of either organ. The chest X-ray was also normal. Repeat laboratory data showed the WBC count to be 3,200, platelets 90,000, uric acid 5.9, total bilirubin 1.2, amylase <30, LDH 18,574, AST 5,498, and ALT 2,254. Gram stain of the amniotic fluid was returned as negative for organisms and WBCs. The differential diagnosis was expanded to include possible cholangitis. The patient was started on intravenous metronidazole for coverage of anaerobic bacteria. A gastroenterological consultation was obtained. The impression was that the patient had common bile duct stones with cholangitis, rule out pancreatitis, rule out hepatitis. IgG TORCH titers were returned with a positive titer for cytomegalovirus. IgM titers were not returned initially. All bacterial cultures were negative.

The patient remained on continuous fetal monitor. Approximately 30 h after admission, the patient developed persistent late decelerations. There were no uterine contractions noted by the patient; however, contractions were picked up by the fetal monitor. The patient was treated with 3 doses of subcutaneous terbutaline but persisted in having contractions. A cesarean section was planned due to the persistent late decelerations. A coagulation survey and CBC were obtained prior to surgery. Platelets were 27,000 and PT and PTT were both markedly elevated. Bleeding time was now 10 min. The patient was transfused with 2 units of fresh frozen plasma and 10 units of platelets. An emergency cesarean section was performed under general anesthesia with delivery of a viable female infant weighing approximately 1,800 g, Apgar 6/8. The placenta showed evidence of retroplacental hematoma consistent with placental abruption.

Postpartally, the PT and PTT remained elevated. Fibrinogen was 177 and platelets 53,000. Four hours postoperatively, the patient began to have a large amount of vaginal bleeding. The uterine fundus was firm on examination. Repeat coagulation studies showed prolonged PT and PTT, fibrinogen of 90, and platelets of 40,000. The patient was transfused with 10 units of platelets and 1 unit of fresh frozen plasma. She became disoriented and developed labored respirations. Arterial blood gases showed a pH of 7.27, $PCO_2$ of 18, $po_2$ of 89, bicarbonate of 8, oxygen saturation of 97% with base excess of 16.3.

The impression at this time was metabolic acidosis secondary to sepsis vs. hepatitis. Antibiotics were continued. The patient was transfused with 8 units of packed RBCs, 9 units of fresh frozen plasma, and 10 units of platelets, but continued to bleed profusely. Twenty-four hours postoperatively, the patient became unresponsive to pain, and her urine output dropped to 0. She experienced a grand mal seizure and her pupils were noted to be fixed and dilated. Two successive electroencephalograms were read as flat line. The patient's ventilator support was removed on December 27, 1991. TORCH IgM titers were returned only after the patient had become unresponsive and were found to be positive for herpes simplex virus (HSV) type I and type II. A repeat HIV was negative. Epstein-Barr virus IgM was also negative. The patient was negative for leptospirosis.

An autopsy was performed and was limited to the thorax and abdomen at the family's request. Autopsy findings were consistent with herpetic hepatitis with fulminant hemorrhagic hepatitis necrosis. Figure 1 demonstrates hepatic necrosis with

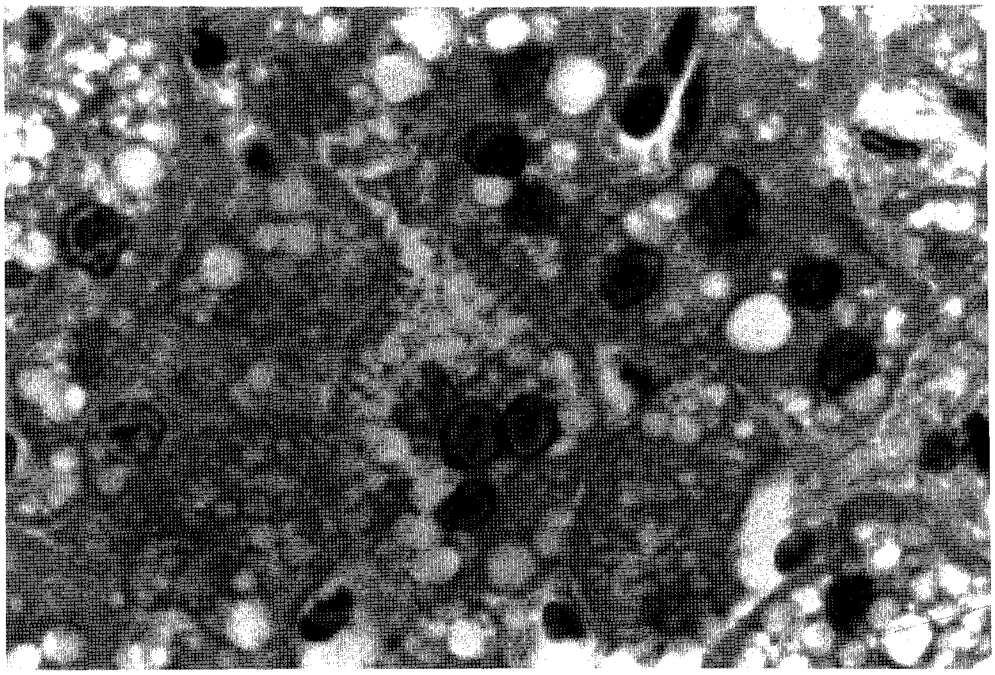

**Fig. 1. Hepatic necrosis with herpetic inclusions.**

herpetic inclusions. Herpetic inclusions were also identified in the endocervical glandular epithelium.

### DISCUSSION

Fourteen cases of herpetic hepatitis have been reported in the literature.[1] Herpetic hepatitis is a difficult diagnosis to make because of confusion with pregnancy-specific hepatic disorders, such as HELLP syndrome (hemolysis, elevated liver function tests, low platelets) and acute fatty liver of pregnancy. Herpetic hepatitis is extremely rare, with a maternal mortality rate of 43%.[1] The high mortality rate may be due to a defect in the cell-mediated response in the pregnant patient.

There are several distinguishing characteristics of herpetic hepatitis as stated in the literature. These include onset of disease in the 3rd trimester, a prodromal illness, vulvar or oropharyngeal vesicular lesions, primary HSV infection, and anicteric presentation. The patient in this case had the above presentations; however, she had no obvious vesicular lesions. The postmortem examination did show evidence of an endocervical herpetic infection.

Of the 14 patients reported in the literature, 6 of them died. Four patients treated with parenteral acyclovir survived and 2 of the patients treated with the antiviral agent vidarabine also survived; however, 5 of 7 patients who did not receive antiviral therapy died. Parenteral acyclovir has been shown to be efficacious in the treatment of disseminated HSV. Not only has acyclovir improved maternal morbidity rates, but it also has been shown to improve neonatal HSV infection.[1,2]

Herpetic hepatitis is a difficult diagnosis to make. Disseminated herpes has a high mortality rate in pregnant patients.[1,2] It is essential to make a diagnosis in order to institute therapy. HSV hepatitis should be included in the differential diagnosis of hepatic dysfunction in the 3rd trimester. The pregnant patient with a primary HSV lesion should be closely watched for any evidence of disseminated disease. Diagnosis can be made by liver biopsy to confirm the presence of herpetic intranuclear inclusion bodies. Once the diagnosis is made, acyclovir can be initiated to significantly reduce maternal mortality.[1]

### REFERENCES

1. Klein NA, Mabie WC, Shaver DC, Latham PS, Adamec TA, Pinstein ML, Reilly C: Herpes simplex virus hepatitis. Gastroenterology 100:239–244, 1991.
2. Wertheim RA, Brooks BJ, Rodriquez FH, Lesesne HR, Jennette JC: Fatal herpetic hepatitis in pregnancy. Obstet Gynecol 62:38–42, 1983.

## References

[PDF_00217] Klein N. A., Mabie W. C., Shaver D. C., Latham P. S., Adamec T. A., Pinstein M. L., Riely C. A. (1991). Herpes simplex virus hepatitis in pregnancy. Two patients successfully treated with acyclovir.. Gastroenterology.

